# Uterine irradiation as a determinant of infertility and pregnancy losses in young cancer survivors

**DOI:** 10.3332/ecancer.2020.1032

**Published:** 2020-05-06

**Authors:** Barbara Buonomo, Roberto Orecchia, Federica Tomao, Lino Del Pup, Alex Garcia-Faura, Fedro A Peccatori

**Affiliations:** 1 Fertility and Procreation Unit, Division of Gynaecologic Oncology, European Institute of Oncology IRCCS, Milan, Italy; 2 Scientific Directorate, European Institute of Oncology IRCCS, Milan, Italy; 3 Department of Gynaecologic Oncology, European Institute of Oncology, IRCCS, Milan, Italy; 4 University Sanitary Agency Friuli Central (ASU FC) Italy; Board Italian Society of Third Age Gynaecology (SIGiTE), Italy; 5 Oncofertility Unit, Institut Marquès, Barcelona, Spain

**Keywords:** uterine irradiation, radiotherapy, cancer survivors, pregnancy outcome, infertility

## Abstract

Several concerns exist regarding the impact of anticancer treatments on fertility and pregnancy outcome. The detrimental effects of both chemotherapy and radiotherapy on the ovaries are well reported in the available literature. Fewer data are focused on the importance of a functioning uterus to conceive and carry on a healthy pregnancy. The aim of this paper is to provide a narrative review of the current literature to assess the role of uterine irradiation as a potential determinant of infertility and poor obstetrical outcomes. This review addresses the need for multidisciplinary counselling in order to face the poor reproductive and obstetrical outcomes of women who had uterine radiation, according to the different backgrounds (radiotherapy during adulthood versus childhood; total body irradiation versus pituitary, spinal and/or abdominal-pelvic irradiation).

## Introduction

Nowadays, cancer survival continues to increase across high-income countries, even if international disparities persist [1]. Moreover, there is a rising trend of delaying childbearing for personal, educational and professional reasons. Given that cancer incidence increases with age, more women enquire about the feasibility and safety of pregnancy following a cancer diagnosis. Additionally, several concerns exist regarding the impact of cancer therapy on the pregnancy outcome [2]. Anderson *et al* [3] reported a standardised incidence ratio of 0.62, indicating that the number of pregnancies in a large cohort of cancer survivors is much lower than expected in the general population [3]. This data could be explained by a loss of the reproductive function potentially induced by the need of chemotherapy, radiotherapy (RT) or a combination of both [4].

A successful pregnancy depends on the integrity of the hypothalamic-pituitary-ovarian axis, oocyte reserve and quality, and on a uterine environment that is not only receptive to implantation, but also able to allow a normal foetal growth. Hence, adverse effects of anticancer treatments on the reproductive function and on pregnancy outcome may be mediated through one or more parts of the reproductive system. Focusing on RT, it’s well established that it could result in ovarian injury: half of the total number of nongrowing follicles are lost at doses of 2 Gy (LD50) [5], which is much lower than the dose delivered in a curative setting. The patient’s age, the total dose, the dose per fraction and the volume of irradiation fields are the most important determinants of the extent of the damage in terms of degree of follicular loss and risk of premature ovarian failure (POF). The biological response to radiation varies also with the number of fractions delivered. For example, a single dose to the testis may be less damaging to the germinal epithelium than the same dose received over several fractions, whereas the reverse is true for the ovary [6]. Total body irradiation (TBI) (9.2–15.75 Gy) results in POF in 90% of patients in long-term follow-up [7]. After whole abdominal radiation (20–30 Gy), ovarian failure rates may be as high as 97% [7].

Ovarian transposition is an option to preserve the ovarian function for patients who are candidates for pelvic irradiation. Placing the organ outside the radiation field (e.g., laterally and above the pelvic brim) is considered as safe and effective; however, this procedure could fail because of the radiation scatter or remigration of the transposed ovaries [8]. Moreover, due to the wider availability of assisted reproductive technology (ART), there are several strategies of fertility preservation, as controlled ovarian hyperstimulation and oocyte or embryo cryopreservation prior to chemoradiation. Nevertheless, for those women who subsequently use their own stored oocytes or embryos or for those who undergo egg-donation to achieve a pregnancy, there is very little available evidence whether the irradiated uterus can successfully and safely carry a pregnancy. Specifically, implantation and on-going pregnancy (defined as detectable gestational sac at 12 weeks) rates were significantly lower in cancer survivors than in control women, also with ARTs [9–11]. Van der Ven *et al* [12] reported no pregnancies in five women who have previously received radiotherapy to the pelvis after transplantation of cryopreserved ovarian tissue, despite the resumption of regular menstrual cycles [12]. Thus, women facing treatments including pelvic RT at doses higher than 40–45 Gy are usually excluded from fertility preservation programs, because the sterility caused by uterine irradiation is considered not treatable. Prepubertal girls are more vulnerable and dose of >25 Gy in childhood focused directly to the uterus induces irreversible damage. Facing this question is a main critical issue in the field of oncofertility.

The aim of this paper is to provide an overview on uterine irradiation as a potential reason of infertility and negative obstetrical outcomes in young cancer survivors.

## Methods

We performed a narrative review of the literature on PubMed (Medline), with restriction to publications in English, using the following keywords: uterine irradiation, radiotherapy, cancer survivors, pregnancy outcome and infertility. There were no limits placed on time of publication. We included all the cases in which the uterus is directly or indirectly involved in the radiation field (TBI, pituitary, spinal and/or abdominal-pelvic irradiation).

## Mechanisms and determinants of uterine radiation-induced damage

The first evidence that RT may damage the uterus emerged in 1989 when Wallace *et al* [13] described the reproductive outcomes for a cohort of 38 young women who received high dose abdominal or flank irradiation for childhood cancer treatment [13]. While most of them failed to enter puberty or developed POF, four women became pregnant in their early twenties, but they all experienced second trimester miscarriages. There was no mention of foetal abnormalities that may have induced miscarriage, but laparoscopy showed that one patient had an atrophic uterus. The authors suggested that the fibrosis of the uterus following radiation exposure may have resulted in pregnancy losses.

Uterine damage could be subsequent to the involvement of different tissues: the endothelium of uterine vessels, the myometrium, the endometrium and/or pelvic floor muscles. At first, radiation-induced fibrosis and obliterations of myometrial and endometrial vessels implicates a decreased uterine blood flow. Noteworthy, an increased uterine artery pulsatility index was reported in cancer patients who underwent TBI before a bone marrow transplant [14]. RT can also damage muscle fibres and decrease pelvic floor muscle function.

Ultrasound (US) and magnetic resonance imaging (MRI) may be very useful tools to detect changes in uterine tissues since 1 month after the last RT cycle: diagnostic imaging shows a reduced uterine volume due to a sclerotic myometrium, a thinner endometrium and a decreased uterine blood flow. These modifications lead to an impaired uterine elasticity and trophism and they seem to be more evident after a direct uterine irradiation or when RT was performed before menarche or within 7 years after it [15–18]. These data were confirmed by Critchley *et al* [18] that reported a shorter uterine length and an undetectable uterine blood flow in in women exposed to whole abdominal irradiation during childhood than in patients with adult onset POF an no history of RT.

Most of the available data about this topic comes from the setting of anticancer treatments during childhood or adolescence. There is a lack of evidence among women exposed to uterine irradiation during adulthood. One of the most relevant paper focused on uterine changes after pelvic RT at a dosage of 40–65 Gy during adulthood was the one published by Arrive *et al* [17]: they described a significant myometrial alteration on T2-weighted MRI, just 1 month after the completion of RT. This resulted in a reduction of the uterine volume at least 3 months after the radiation treatment, while a diminished endometrial thickness was shown after 6 months [17].

As for the ovarian injury, uterine radiation-induced damage depends not only on patient’s age, but also on the total radiation dose and on the site of irradiation.

There are also very limited data on the critical dose for endometrial function. It is assumed that a uterine radiation dose <4 Gy does not impair the uterine function, whereas doses between 14 and 30 Gy have been reported to result in uterine dysfunction [7, 19]. A dose > 45 Gy during adulthood (>25 Gy during childhood) probably is incompatible with further pregnancy [15].

RT for several neoplasms (i.e., haematological, cervical, anal and colorectal cancer, and soft tissue sarcomas) often has to involve the uterus, partially or whole, in the clinical target volume [20, 21]. Also some precancerous and/or non-malignant diseases, such as myelodysplasia, aplastic anaemia, thalassemia and systemic lupus erythematosus, could need high-dose gonadotoxic chemotherapy with or without TBI. Abdominal and pelvic irradiation may be part of the management of Wilms tumour, pelvic rhabdomyosarcoma, and Ewing sarcoma of the pelvis or spine [22].

Larsen *et al* [16] showed that patients who have received direct uterine irradiation had significantly lower uterine volume than those who had not received RT or those who underwent RT to field above or below the diaphragm (*p* < 0.02 in all comparisons). No statistically significant difference was found in uterine volume between patients treated with RT below the diaphragm and patients who had not received RT; however, some patients irradiated below the diaphragm (but not directly to the uterus) had very small uterine volume suggesting that scattering radiation might have occurred.

More recently, Van de Loo *et al* [23] evaluated the effects of uterine irradiation in a large population of childhood cancer survivors (CCSs), and compared outcomes with those of a group of nonirradiated CCSs and a group of women from the general population. CCSs enrolled in this study underwent RT to a field that certainly (TBI; pelvic RT) or most likely (low-abdominal and lower spinal RT) involved part of the uterus. The median uterine volume did not seem to be significantly smaller in RT-exposed CCSs compared with non-RT-exposed CCSs. As expected, RT-exposed CCSs resulted to be at increased risk of a reduced uterine volume (<44.3 ml) compared with controls from general population. Remarkably, the same was true for non-RT-exposed CCSs compared with controls. This means that not only uterine irradiation, but also chemotherapy could play an important role [24]. Anyway, only one paper showed an impaired uterine volume in patients treated with an alkylating agent (busulfan) [14] as pre-conditioning treatment for bone marrow transplant.

## Reproductive and obstetrical outcomes after uterine irradiation

Reinmuth *et al* [25] reported the analysis of the first nationwide survey among German adults with a history of childhood cancer; they showed that pelvic irradiation strongly affects fertility [25]. RT-induced injury in the glandular and stromal components of the endometrium causes destruction of progenitor basal cell layer and consequent subfertility or infertility by inducing a state of endometrial atrophy that could forbid the embryo implantation [15]. CCSs are more likely to suffer from clinical infertility and have prolonged time to achieve than their siblings even when they retain ovarian function after exposure to radiation [26].

Even if a pregnancy occurs, population-based studies of cancer survivors have demonstrated an association between TBI, abdominal and/or pelvic RT and adverse pregnancy and neonatal outcomes, including placental abnormalities, early pregnancy loss, preterm birth, low birthweight (LBW) infants (<2,500 g) and perinatal mortality [23, 27–33]. Reduced uterine volume, impaired uterine elasticity, and damage to the uterine vasculature, and not oocyte damage, have been suggested to cause these poor obstetrical outcomes [34]. Of them, uterine volume reduction may play a central role [16]. Larsen *et al* [16] reported that mid-trimester miscarriages occurred significantly in patients who received RT at a younger age and directed to fields below the diaphragm and to fields directly involving the uterus (*p* = 0.007): of 16 patients with previous uterine irradiation, six became pregnant, of which three had a mid-trimester abortion, one a preterm birth, and two had term deliveries. Miscarriage occurred in those with a uterine volume of 20–40 mL. No women irradiated under 8 years of age and with a uterine volume < 20 mL became pregnant.

Conversely, few case reports of successful pregnancies were published in women exposed to high doses of pelvic radiation during adulthood [7, 35–37].

Signorello *et al* [30] reported that uterine exposure to RT was associated with increased risk of preterm birth. The damage was greater, and the threshold lower, for patients treated before menarche. The severity of preterm delivery is also reported to increase with the dose of irradiation [29, 30]. High-dose uterine irradiation was also associated with children born small when compared with others of the same gestational age and, furthermore, with LBW. No statistically significant differences between groups were found regarding the probability of having small for gestational age offspring or a miscarriage. This implies that RT directed to the abdominal-pelvic area impairs the uterine ability to sufficiently expand and carry a pregnancy to term rather than impair placental function [23, 29, 38]. The mechanisms by which RT could cause preterm delivery are speculative, but several possibilities exist. First, the physical constraint of the pregnancy induced by somatic effects, including decreased uterine volume, as described above, could influence the risk of preterm birth. Also, uterine fibrosis might impair cervical competence or placentation (leading to abruptio placentae), both factors that are linked to preterm birth [30, 39]. An excess of fetal malpresentation is reported among female cancer survivors treated with flank irradiation [29], and malpresentation is a known risk factor for preterm birth [40].

Signorello *et al* [41] reported that uterine or ovarian irradiation was also associated with an increased risk of stillbirth or neonatal death, that seemed to be independent from the risk of preterm birth, suggesting that RT has a role in the cause of late foetal death [41]. They could not directly establish whether uterine injury or oocyte damage was the cause of the association with stillbirth or neonatal death, but a uterine effect was most likely. A small proportion of patients with Wilms’ tumours included in this study might also have uterine anomalies that could contribute to the increased risk of stillbirth or neonatal death, independently of radiation effects [42]. Women who had a successful pregnancy with a past history of RT usually require caesarean section [43, 44].

In the British Childhood Cancer Survivor Study, a threefold risk of gestational hypertension was described for survivors of Wilms tumour treated with abdominal RT [44]. The rate of gestational diabetes is not consistently higher in cancer survivors than in controls [38], but abdominal RT has been associated with a 2.7- to 4.7-fold higher risk in one study [31, 45].

## Potential strategies to preserve/improve the uterine function

Azaïs *et al* [46] described uterus fixation to the ventral abdominal wall as a safe and feasible surgical technique that could be performed in order to spare as much as possible of the endometrium in case of pelvic RT, in particular for colon-rectal cancer [46]. However, a cost-effectiveness analysis of this procedure is lacking and future research is necessary.

Moreover, the advances in RT treatment planning such as intensity-modulated radiation therapy, image-guided radiation therapy and proton therapy techniques offer the possibility to administer high doses to a more conformal volume while sparing organs at risk as the uterus [47].

The efficacy and the most appropriate dose, formulation and route of administration of sex hormone replacement therapy (HRT) to young women with ovarian failure after pelvic irradiation have not yet been established. No improvement in endometrial thickness and blood supply was observed after 1 month of HRT in three survivors with ovarian failure after treatment with abdominal irradiation [19]. Larsen *et al* [16] did not demonstrate significant improvement in uterine volume, endometrial thickness or uterine blood supply in response to a 3-month exposure to high-dose estradiol regimen (percutaneous estradiol 150 μg/24 h) in patients with ovarian failure and small uterine volume following abdominal or pelvic RT [16]. Bath *et al* [48] showed improvement in all uterine characteristics in four women who had received TBI in childhood when HRT was extended over three cycles. An explanation may be that abdominal and pelvic irradiation (30–45 Gy) probably causes more damage to uterine musculature and vasculature compared with TBI (14.4 Gy). Bath reported that even if uterine volume improved, it remained significantly lower in girls exposed to RT than in controls, particularly in those treated with RT before puberty [48]. In the paper by van de Loo *et al* [23], the use of HRT (oral and intrauterine device) did not significantly change the uterine volume.

These data are important, because the lack of a consistent uterine response even after 3 months of high-dose estradiol impairs the possible success of egg-donation in women who have received abdominal or pelvic irradiation.

Encouraging results have been reported with the use of HRT together with the antifibrotic agents pentoxifylline (PTX) and tocopherol (vitamin E) [49]. The combined use of PTX and tocopherol seems to induce regression of radiation-induced fibrosis both in animal and human studies [49].

The advent of uterus transplantation may change the scenario but, to date, counselling patients based on this opportunity is not feasible [50]. Another alternative could be surrogacy that is currently banned in several countries.

## Conclusions and take-home messages

In conclusion, cancer survivors who were exposed to uterine irradiation and are interested in childbearing should be counselled by a multidisciplinary team involving the medical or paediatric oncologist, the radiation oncologist, the gynaecologist, and the reproductive endocrinologist. A simplified list of take-home messages is included.

1. Uterine irradiation could damage the myometrium, the endometrium and the uterine vasculature leading to smaller and less elastic uterus. The extent of this injury depends on patient’s age, field of irradiation and total radiation dose.
2. Cancer survivors previously exposed to RT that involved the uterus are at increased risk of negative reproductive and obstetrical outcomes, such as infertility, mid-trimester miscarriage, preterm birth, LBW infants, stillbirth and neonatal death. This is particularly relevant in women who were irradiated before menarche.
3. Since the decreased uterine volume may be the main determinant of pregnancy complications, and since the volume cannot be significantly improved by HRT, uterine size (assessed by ultrasound or MRI) may determine the pregnancy outcome.
4. Little evidences are available about reproductive and obstetrical outcome of women who had pelvic radiation during adulthood, but some case reports of successful pregnancies after adulthood RT have been described [7, 35–37].5) HRT ± PTX-Vit E could improve the uterine environment, even if data are not conclusive or particularly encouraging.
5. Caesarean delivery should be advised in most cases because of the increased risk of uterine rupture, placental anomalies and foetal malpresentation.
6. In case of TBI/partial pelvic irradiation at a dose of 4–45 Gy (4–25 Gy if childhood radiation), patients should be aware that pregnancy might occur, but the risk of pregnancy complications is increased.
7. In case of whole pelvic radiation at a dose > 45 Gy (> 25 Gy if childhood radiation), pregnancy should be discouraged and contraception advised.

## Conflicts of interest

The authors declare that they have no conflicts of interest.

## Funding statement

No funding was received for this specific research.

## Authors’ contributions

All authors listed have contributed sufficiently to the writing and/or critical revision of the paper; they have approved the submitted final version.
